# HIV/AIDS stigma accumulation among people living with HIV: a role of general and relative minority status

**DOI:** 10.1038/s41598-023-37948-7

**Published:** 2023-07-03

**Authors:** Ewa Gruszczyńska, Marcin Rzeszutek

**Affiliations:** 1 Faculty of Psychology, SWPS University of Social Sciences and Humanities, Chodakowska 19/31, 03-815 Warsaw, Poland; 2 Faculty of Psychology, University of Warsaw, Stawki 5/7, 00-183 Warsaw, Poland

**Keywords:** Psychology, Human behaviour

## Abstract

The main objective of the study was to investigate the relationship between selected sociodemographic factors (i.e. sexual orientation, gender and AIDS status), and the level of HIV/AIDS stigma among people living with HIV (PLWH). The participants were 663 adults with a medically confirmed diagnosis of HIV infection, undergoing antiretroviral treatment. Their level of HIV/AIDS stigma was assessed with the Berger HIV Stigma Scale, and relevant sociodemographic and clinical data were obtained using a self-report survey. The main effect was revealed only for sexual orientation and total stigma; those with heterosexual orientation declared higher levels of total stigma than those with other sexual orientations. For the subscales, significant results were obtained only for disclosure concerns. Namely, for the interaction of gender and sexual orientation, the highest level of disclosure stigma was declared by heterosexual women, while there was no such relationship for men. This result was further modified when AIDS diagnosis was added to the interaction. There is a cumulative effect of PLWH minority statuses, rather than main effects of each status individually. Thus, each minority status should be analysed from at least two perspectives, general (i.e., compared to the general population) and relative (i.e., compared to the population in question).

## Introduction

June 2021 marked the fortieth anniversary of the first cases of human immunodeficiency virus (HIV) infection being detected by the Centers for Disease Control and Prevention^1^, which initiated the acquired immunodeficiency syndrome (AIDS) pandemic. Over this time, great medical progress in HIV treatment has changed HIV/AIDS from a death sentence to a chronic and manageable health problem^2^. This transformation is highlighted by the fact that the average life expectancy of people living with HIV (PLWH) today does not substantially differ from that of the general population^3^, and the medical status of HIV infection no longer poses the most important predictor of quality of life among PLWH^4^. However, PLWH still experience intense HIV-related distress and consistently report lower levels of quality of life in comparison not only with the general population but also with patients suffering from other chronic illnesses (e.g., rheumatoid arthritis and diabetes mellitus types 1 and 2)^5^. This pessimistic trend is associated with the present stigmatisation of PLWH. Although the explicit manifestations of this have altered, the overall level of stigmatisation remains rather similar to what it was at the beginning of the HIV/AIDS epidemic^6,7^. In fact, HIV/AIDS stigma is treated as the main source of psychological distress and low health-related quality of life for PLWH, as well as the greatest barrier to effective coping with the HIV epidemic in healthcare worldwide^3,8^

Stigma can be conceptualised from two different but related perspectives^9^. At the societal level, it is defined as the negative perception of a particular trait or characteristic by others^10^. In the context of HIV, stigma encompasses negative attitudes, behaviors, and judgments directed towards individuals living with or at risk of HIV. Generally, HIV stigma originates from a fear of HIV, often influenced by the initial images associated with HIV that emerged in the early 1980s^11^. The misconceptions created then about HIV transmission, treatment options and the functioning of PLWH persist to this day. According to UNAIDS^12^, HIV stigma significantly hampers the HIV response by impeding access to prevention services, sexual and reproductive health services, as well as testing, treatment, and adherence. At the individual level, as outlined by Earnshaw and Chaudoir^13^, stigma operates through three mechanisms. Enacted stigma refers to the experiences of PLWH who have encountered prejudice and discrimination from others. Anticipated stigma pertains to the expectation held by PLWH that they will encounter prejudice and discrimination from others in the future. Internalized stigma, on the other hand, encompasses the negative beliefs and attitudes that PLWH have about themselves as a result of being infected with HIV.

A huge number of studies have been conducted to understand the complex process of PLWH’s stigmatisation, which encompasses both the internal traumatic character of HIV/AIDS itself as a potentially infectious and life-threatening condition and the external socio-cultural issues that reveal existing inequalities in class, race, gender and sexuality^6,14^. Regarding this conceptual complexity, psychological research on HIV/AIDS stigma is still searching for an empirically validated theoretical model that could provide a clear definition of the term and identify the processes through which stigma worsens the quality of life of PLWH^15,16^. Moreover, to fully understand the mechanisms and effects of HIV/AIDS stigma, it is vital to also include the minority stress theory, which describes the uniqueness of stressors not only experienced by sexual minorities^17^ but also other stigmatised groups in society, including PLWH^18^. More specifically, it has been observed that HIV/AIDS stigma may be intensified among sexual minorities (e.g. lesbian, gay and bisexual PLWH), who are additionally significantly affected by the HIV epidemic^19–22^. Therefore, among PLWH, the following two levels of stigmatisation are observed: one related to being diagnosed with HIV (i.e. objective medical status defined by a specific social construction and reception), and the other related to being in a sexual minority. Obviously, not every person infected with HIV is in a sexual minority, but those who are may be prone to stigma accumulation^22^. It has been documented in several studies that PLWH who are in a sexual minority have even lower well-being and worse health than the general population of HIV/AIDS patients^23^. The minority stress theory explains that this as a result of their disproportional exposure to stigma-related stress due to their double devaluated social status^17^. The matter  is further complicated by the fact that the process of stigma accumulation has been found to  be more pronounced not only among females infected with HIV, compared with HIV-infected males^24^, but also among PLWH in the AIDS phase, with visible signs of HIV infection, in comparison with PLWH with good medical control of their infection^6,25^. Thus, being infected with HIV can result in multiple sources of stigma-related stress, depending on availability of minority identities in a specific sample^7,18^.

This research area held significant importance during the COVID-19 pandemic, which presented a global challenge for mental health worldwide^26^. It holds particular significance among various marginalized populations, including PLWH^27^. PLWH, specifically, faced numerous disruptions to their daily lives, such as obstacles to healthcare due to COVID-19-related hospital changes, delays in HIV testing, difficulties accessing HIV treatment, and compromised privacy due to telemedicine services^28^. Additionally, being infected with HIV turned out to be an independent risk factor for both severe COVID-19 at admission and in-hospital mortality^29^. Furthermore, PLWH faced additional isolation and stigmatization resulting from misinformation surrounding a perceived connection between COVID-19 vaccines and the risk of HIV infection^30^. These factors could have amplified the already high levels of HIV/AIDS stigma and societal fears towards PLWH, particularly those from gender and sexual minorities^27^.

### Current study

Taking the above-mentioned research gaps into an account, the main purpose of this study is to investigate the relationship between selected sociodemographic factors (i.e. sexual orientation, gender and AIDS status) as potential sources of stigma accumulation and the level of HIV/AIDS stigma and its subscales (see Measures) among a sample of PLWH. In particular, we seek to verify if participants’ sexual orientation, gender and AIDS status act as independent and interacting vulnerability factors. We formulated the following three research hypotheses:

#### *Hypothesis 1*

There are significant main effects of participants’ sexual orientation, gender and AIDS status on their level of HIV/AIDS stigma. That is, participants whose sexual orientation is other than heterosexual, who are women and who have been diagnosed with AIDS have a higher intensity of HIV/AIDS stigma compared with participants who are heterosexual, men and not diagnosed with AIDS status, respectively. These effects are present for both stigma subscales and overall stigma level.

#### *Hypothesis 2*

There are significant two-way interactions between sexual orientation, gender and AIDS status on the level of HIV/AIDS stigma. Specifically, the effect of one minority status is further exacerbated by another minority status; that is, having two minority conditions together leads to HIV/AIDS stigma that is higher than each condition separately. These effects are present for both stigma subscales and overall stigma level.

#### *Hypothesis 3*

There is a significant three-way interaction between sexual orientation, gender and AIDS status on the level of HIV/AIDS stigma; that is, having three minority conditions leads to HIV/AIDS stigma that is higher than when there are two such conditions. This effect is present for both stigma subscales and overall stigma level.

## Method

### Participants and procedure

The study sample was recruited from the outpatient clinic of a state hospital of high reference for the diagnosis and treatment of infectious diseases. We adopted the following eligibility criteria: 18 years of age or older, medical diagnosis of HIV infection and undergoing antiretroviral treatment in the clinic where the study was conducted. Regarding the exclusion criteria, patients with HIV-related cognitive disorders or current abuse of psychoactive substances, as determined by medical doctors, were not included in the study. The doctors provided the relevant information, simplified as either *eligible* or *not eligible* for the study, directly to the research assistants. Access to medical records was not permitted due to ethical reasons and legal protection of medical data.

Adopting these criteria resulted in 664 participants who provided informed consent to take part in this study. The measurement was conducted between July and October 2020, during the so-called ‘first wave’ of the COVID-19 pandemic. Participants completed a paper or online version of the questionnaires and participated in the study voluntarily without remuneration. The protocol of the study was accepted by the local ethics commission. One person marked ‘other’ in response to gender and was not included in further analysis. The final sample thus consisted of 663 PLWH, and their basic sociodemographic and clinical characteristics are displayed in Table 1. The results of cross tabulation of minority statutes informs that women declared mainly being heterosexual (75.0%), whereas men declared being mainly other than heterosexual (89.3%), Yule’s Phi = 0.55, *p* < 0.001; women were more often diagnosed with AIDS (22.6%) than men (13.7%), Yule’s Phi = − 0.08, *p* < 0.05; and participants with heterosexual orientation were more often diagnosed with AIDS (24.8%) than participants with other sexual orientations (12.5%), Yule’s Phi = − 0.14, *p* < 0.001.

**Table 1:** Sociodemographic and clinical characteristics of the studied sample (n = 663).

Variable	n (%)
Gender	
Man	579 (87.3%)
Woman	84 (12.7%)
Age in years (M ± SD)	39.64 ± 9.96
Sexual orientation	
Heterosexual	125 (18.9%)
Homosexual	434 (65.5%)
Bisexual	66 (10.0%)
Other	38 (5.7%)
HIV/AIDS status	
HIV+ only	363 (84.9%)
HIV/AIDS	98 (14.8%)
Missing data	2 (0.3%)
HIV infection duration in years (M ± SD)	9.37 ± 8.20
Antiretroviral treatment (ART) duration in years (M ± SD)	7.65 ± 6.48
CD4 count	592.65 ± 243.08

M = mean; SD = standard deviation.

### Measures

#### HIV/AIDS stigma

To evaluate overall stigma and its subscales, the Berger HIV Stigma Scale was used^31,32^ in its shorter, 12-item version^33^. The Berger HIV Stigma Scale is the most widely used tool for assessing HIV/AIDS stigma. In its original form, it consists of 40 items grouped into four subscales (personalised stigma, disclosure concerns, negative self-image and concern with public attitudes towards people with HIV), but some items are assigned to more than one subscale at a time, resulting in multicollinearity when subscales are analysed jointly. To avoid such redundancy, we used the abbreviated version, where each item belongs to one subscale only. Thus, we had three items per subscale, all sourced from the original version, and this structure was validated through confirmatory factor analysis, demonstrating a good fit to the data collected in the current sample (CFI = 0.98, TLI = 0.97, RMSEA = 0.04 with 90% CI [0.03, 0.05]; all standardized factor loading values > 0.50 and significant at *p* < 0.05). The answers were provided on a four-point Likert scale (1 = strongly disagree, 2 = disagree, 3 = agree, 4 = strongly agree). The personalised stigma subscale was used to evaluate the personally experienced consequences of other people knowing about an individual’s HIV status. The disclosure concerns subscale was used to assess an individual’s concerns about disclosing their HIV status. The negative self-image subscale was used to report individuals’ negative feelings towards themselves due to HIV. The concern with public attitudes subscale was used to describe how individuals perceive people’s beliefs and feelings about PLWH in general. Overall stigma was calculated as a sum of the scores obtained in all items. The Cronbach’s alpha for overall stigma was 0.85; for the subscales, it was 0.87, 0.75, 0.73 and 0.75, respectively.

#### Minority status

Three potential minority statuses were analysed, namely gender, sexual orientation and AIDS diagnosis. Gender was measured categorically with the following three possible answer options: woman, man and other. Sexual orientation was assessed by choosing one category from four available (heterosexual, homosexual, bisexual and other). Finally, being diagnosed with AIDS was evaluated with a binary yes/no answer. For further analysis, gender was coded as 0 for women and 1 for men; the person who marked the third option, as already described, was excluded from the data because ‘other’ could not be classified in the above two categories and represented an outlier in the sample. Sexual orientation was recoded to ‘heterosexual’ (coded 0) and ‘other than heterosexual’ (coded 1), the latter consisting of the three remaining categories. The current AIDS status was coded 1 for ‘yes’ and 0 for ‘no’.

### Data analysis

A three-way analysis of variance was performed to examine whether the total reported stigma, as well as the subscales, differed by gender, sexual orientation, being diagnosed with AIDS and their interactions. Multivariate analysis of variance (MANOVA) was used to analyse together all the subscales after checking for their multicollinearity, while the total score, being a sum of all these subscales, was analysed separately using univariate analysis of variance (ANOVA). We also checked for assumptions of normality, homogeneity of variance–covariance matrices (the Box’s M test) and linearity (by visual inspection of the scatterplot matrix). Partial eta squared (partial η^2^^34^) was used to assess the effect size for comparative purposes due to the frequency of its reporting. It informs about a proportion of a variance that is explained by a given independent variable from the total variance of a dependent variable, remaining after accounting for the variance explained by other variables in the model. However, this popular measure of effect size is known to have positive bias^35^. Therefore, we also provided partial omega squared (partial ω^2^)^36^ as an unbiased alternative. The interpretation of both these measures are the same with cut-offs of 0.01, 0.06, and 0.14 to indicate small, medium and large effects respectively^37^. For MANOVA, only significant multivariate effects were analysed further, as they take into account the joint distribution of all the dependent variables and control for Type I error without the need for any additional adjustment^38^. In the interactions, simple main effects were compared using estimated marginal means with Bonferroni correction of significance level and confidence intervals. All the analyses were performed with IBM SPSS Statistics (Version 28.0).

### Research involving human participants

This study complies with the Declaration of Helsinki and was performed according to the institutional ethics committee approval (Research Ethics Committee of the Faculty of Psychology, University of Warsaw, decision nr 1/7/2020). Written informed consent was obtained from all participants before participation in the study.

## Results

### Descriptive statistics and missing data analysis

Table 2 presents the descriptive statistics for the total stigma scores as well as for the four subscales. All the variables had a distribution not deviating from a normal distribution. Among the subscales, the highest stigma values were noted for concerns regarding disclosure (mean difference with personalised stigma = 3.52, negative self-image = 3.05, concerns about public attitude = 1.48; all *p*s < 0.001).

**Table 2:** Descriptive statistics of stigma scores.

Variable	Mean	SD	Minimum	Maximum	Skewness	Kurtosis
STIGMA_PS	5.69	2.61	3.00	12.00	0.72	− .0.41
STIGMA_DC	9.21	2.39	3.00	12.00	− 0.69	− 0.25
STIGMA_NI	6.16	2.60	3.00	12.00	0.51	− 0.69
STIGMA_PA	7.73	2.34	3.00	12.00	− 0.12	− 0.64
STIGMA_TOTAL	28.80	7.39	12.00	48.00	0.02	− 0.42

STIGMA_PS = Personalised stigma; STIGMA_DC = Disclosure concerns; STIGMA_NI = Negative self-image; STIGMA_PA = Public attitude concerns; STIGMA_TOTAL = Overall stigma.

As the analysed data were cross-sectional, only up to 3.5% missingness was noted. Additionally, Fisher’s exact test showed no effect of gender (*p* = 0.06), sexual orientation (*p* = 0.17) or being diagnosed with AIDS (*p* = 0.13) on the pattern of missing data.

### Hypothesis testing

For MANOVA, the Box’s M of 72.20 indicated that the homogeneity of covariance matrices across groups was assumed (F(60, 15,307.59) = 1.12,* p* = 0.25). As seen in Table 3, with the use of Wilks’s criterion in multivariate analyses on the combined dependent variables, no significant main effects of gender (Wilks’s Λ = 0.992, F(4, 627) = 1.33, *p* = 0.26), sexual orientation (Wilks’s Λ = 0.991, F(4, 627) = 1.1.47, *p* = 0.21) or being diagnosed with AIDS (Wilks’s Λ = 0.995, F(4, 627) = 0.76, *p* = 0.55) were revealed. Also, the two-way interactions between gender and being diagnosed with AIDS, as well as sexual orientation and being diagnosed with AIDS, were insignificant. Thus, the only significant effects were found for gender × sexual orientation interaction (Wilks’s Λ = 0.978, F(4, 627) = 3.57,* p* = 0.007, partial η^2^ = 0.022, partial ω^2^ = 0.016) and for the three-way interaction, including all minority statuses (i.e. gender, sexual orientation and being diagnosed with AIDS) (Wilks’s Λ = 0.982, F(4, 627) = 2.87, *p* = 0.022, partial η^2^ = 0.018, partial ω^2^ = 0.012).

**Table 3:** MANOVA results: multivariate tests.

Effect	Wilks’s Λ	F_(4, 627)_	*p*	partial η^2^	partial ω^2^
Gender	0.992	1.328	0.258	0.008	0.002
Sexual orientation	0.991	1.473	0.209	0.009	0.003
AIDS diagnosis	0.995	0.758	0.553	0.005	0.000
Gender × Sexual orientation	0.978	3.565	0.007	0.022	0.016
Gender × AIDS diagnosis	0.997	0.404	0.806	0.003	0.000
Sexual orientation × AIDS diagnosis	0.992	1.292	0.272	0.008	0.002
Gender × Sexual orientation × AIDS diagnosis	0.982	2.872	0.022	0.018	0.012

**Figure 1:**
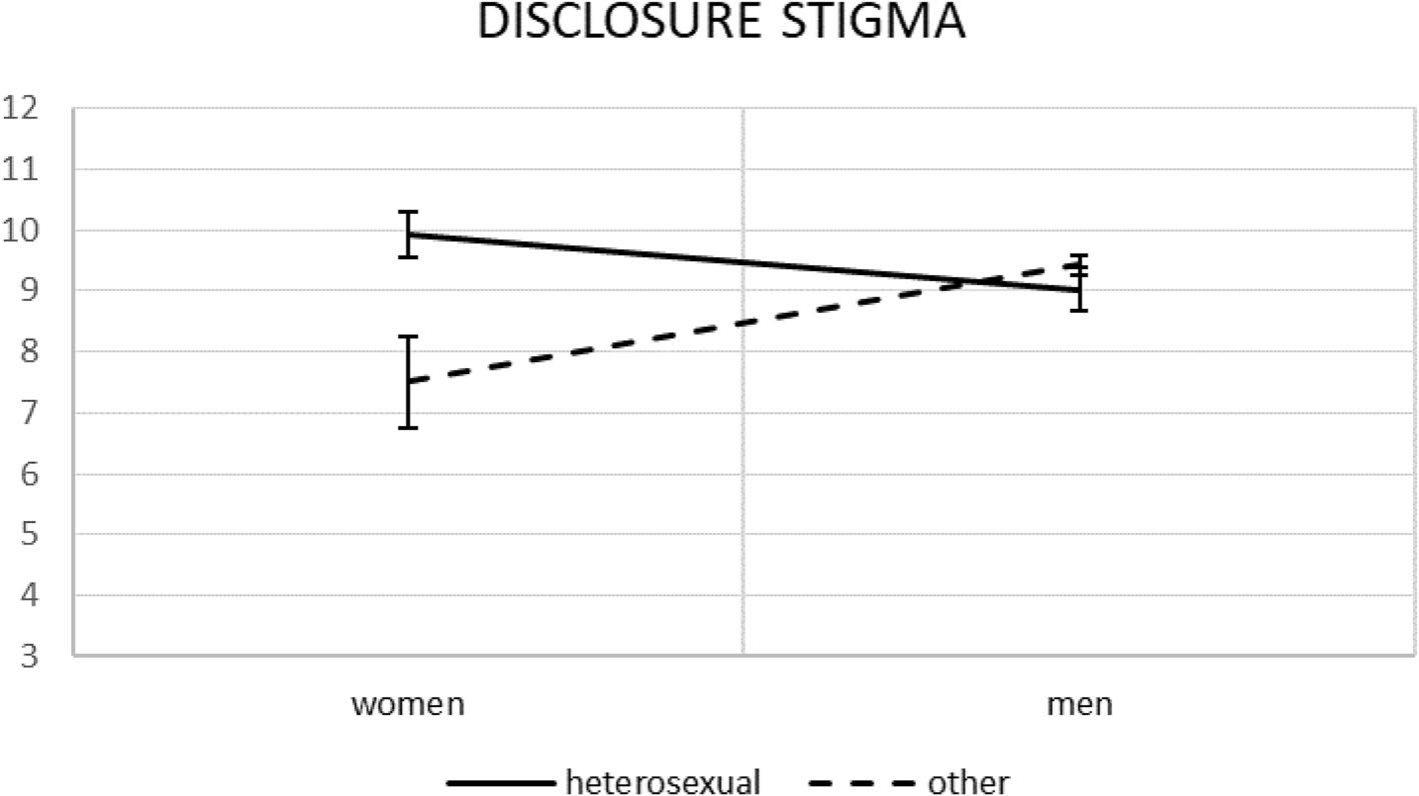
Simple effects for gender × sexual orientation on disclosure stigma with standard errors.

**Figure 2:**
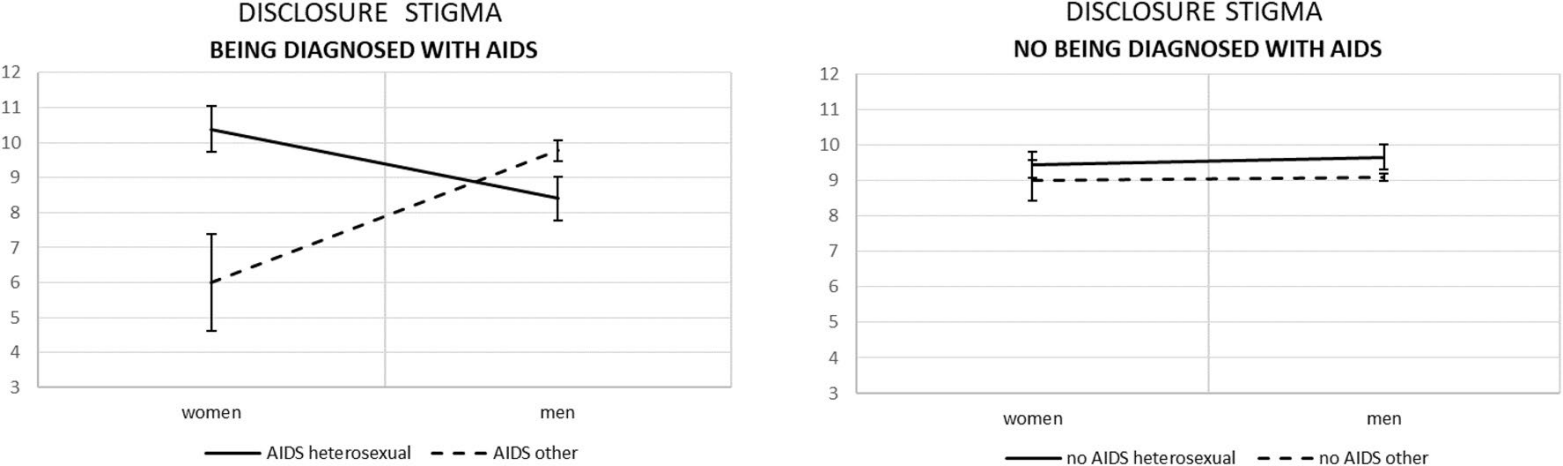
Simple effects for being diagnosed with AIDS × gender × sexual orientation on disclosure stigma with standard errors.

A follow-up analysis of the between-subject effects using a univariate F-test with the Bonferroni correction of an alpha level was performed. It led to a *p* value < 0.0125, which was interpreted as significant. Such results were obtained only for disclosure stigma (see Table 4). The relevant simple effects are plotted at Figs. 1 and 2.

**Table 4:** MANOVA results: tests of between-subjects effects.

Source	Dependent variable	F_(1, 663)_	*p*	partial η^2^	partial ω^2^
Gender × Sexual orientation	STIGMA_PS	4.387	0.037	0.007	0.005
	STIGMA_DC	9.391	0.002	0.015	0.012
	STIGMA_NI	0.006	0.940	0.000	0.000
	STIGMA_PA	5.098	0.024	0.008	0.006
Gender × Sexual orientation × AIDS diagnosis	STIGMA_PS	2.808	0.094	0.004	0.003
	STIGMA_DC	10.305	0.001	0.016	0.014
	STIGMA_NI	0.843	0.359	0.001	0.000
	STIGMA_PA	2.373	0.124	0.004	0.002

STIGMA_PS = Personalised stigma; STIGMA_DC = Disclosure concerns; STIGMA_NI = Negative Self-image; STIGMA_PA = Public attitude concerns.

For gender and sexual orientation, women living with HIV declaring heterosexual orientation reported higher disclosure stigma than those declaring other sexual orientations (mean difference = 2.41, *p* = 0.004, 95% CI [0.77; 4.04]). For men living with HIV, the differences in their sexual orientation were not significant in this regard (mean difference = − 0.40, *p* = 0.30, 95% CI [− 1.17; 0.36]).

Adding being diagnosed with AIDS further modified these results. In particular, there were no significant differences regarding disclosure stigma among participants without AIDS diagnosis, regardless their gender and sexual orientation (see Fig. 2). For women diagnosed with AIDS, the previously noted effect was magnified; that is, heterosexual women diagnosed with AIDS reported higher disclosure stigma than other sexually orientated women diagnosed with AIDS (difference of means = 4.39, *p* = 0.004, 95% CI [1.40; 7.37]). For men diagnosed with AIDS, the previously insignificant effect became significant; those who declared heterosexual orientation reported *lower* disclosure stigma than those who declared other than heterosexual orientation (mean difference = − 1.37, *p* = 0.04, 95% CI [− 2.72; − 0.03]).

**Figure 3:**
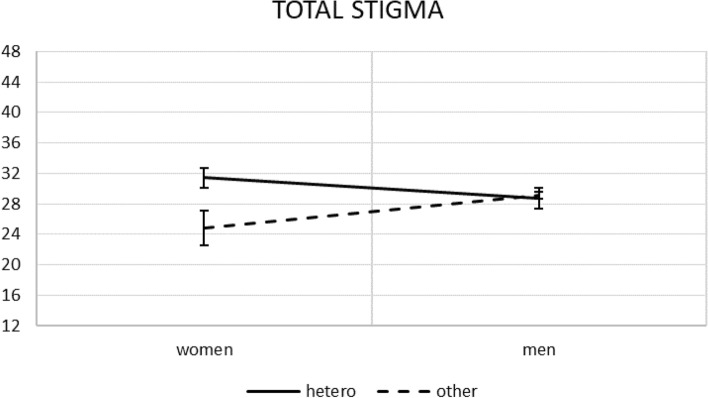
Simple effects for gender × sexual orientation on total stigma with standard errors.

**Figure 4:**
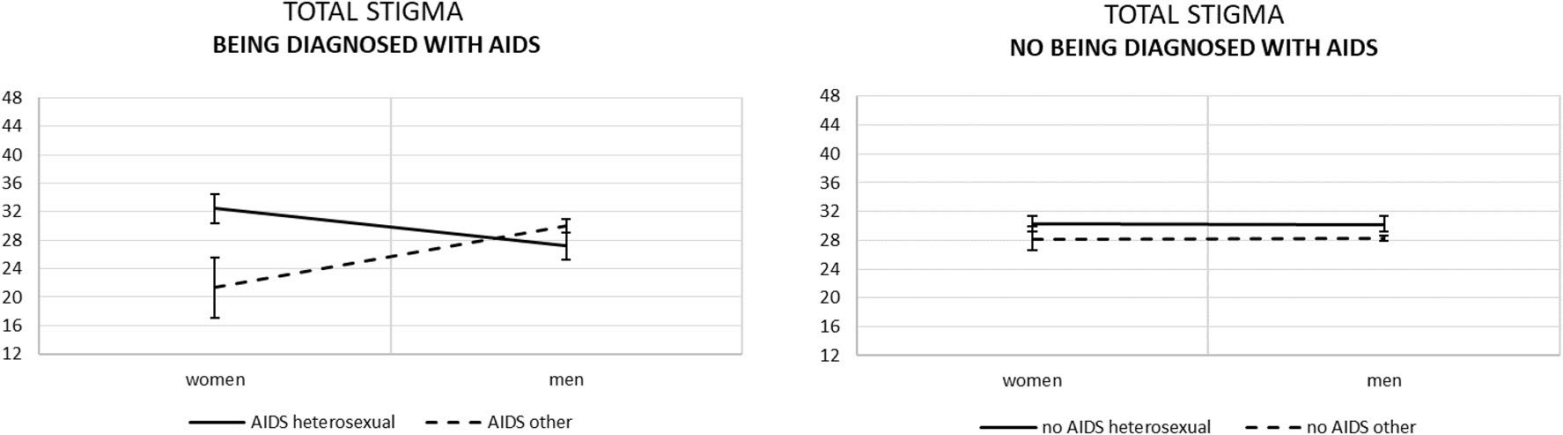
Simple effects for being diagnosed with AIDS × gender × sexual orientation on total stigma with standard errors.

For total level of stigma, analysed in ANOVA, assumption of equality of error variances was confirmed (Levene’s test (7, 630) = 1.21, *p* = 0.297). The results of between-subjects effects are presented in Table 5. Different than for subscales as dependent variables, the main effect was revealed for sexual orientation; that is, PLWH declaring heterosexual orientation reported *higher* overall stigma than those reporting other sexual orientations (30.07 vs. 26.97, mean difference = 3.10, *p* = 0.029, 95% CI [0.32; 5.87]). Also, while the two-way interaction gave the same results as already described for the disclosure stigma (compare Figs. 1 and 3), the three-way interaction brought different results. Thus, as can be seen in Fig. 4, the significant effect was kept only for women diagnosed with AIDS; those declaring heterosexual orientation reported higher total stigma than those declaring other sexual orientations (32.46 vs. 21.33, mean difference = 11.13, *p* = 0.018, 95% CI [1.92; 20.34]).

**Table 5:** ANOVA results: tests of between-subjects effects for total stigma as the only dependent variable.

Effect	F_(1, 630)_	*p*	partial η^2^	partial ω^2^
Gender	0.353	0.553	0.001	0.000
Sexual orientation	4.802	0.029	0.008	0.006
AIDS diagnosis	1.213	0.271	0.002	0.000
Gender × Sexual orientation	6.131	0.014	0.010	0.008
Gender × AIDS diagnosis	0.366	0.545	0.001	0.000
Sexual orientation × AIDS diagnosis	0.579	0.447	0.001	0.000
Gender × Sexual orientation × AIDS diagnosis	5.984	0.015	0.009	0.008

## Discussion

In this study, we analysed three hypotheses regarding the relationship between minority status and stigma, testing both the main effects of each status (i.e. gender, sexual orientation and being diagnosed with AIDS) (Hypothesis 1), the effects of their occurrence in pairs (Hypothesis 2) and all three simultaneously (Hypothesis 3). We separately analysed these effects for the subscales and for overall stigma. The results were marginally consistent for the first hypothesis, as the main effects were only revealed for sexual orientation and total stigma. Moreover, they turned out to be weak and rather opposed to the findings obtained in other studies^19,22^, as not LGBTQ PLWH but heterosexual PLWH were those who declared the higher level of total HIV/AIDS stigma.

Trying to interpret this interesting and nowadays rather counterintuitive result, it should be underscored, however, that in our sample, PLWH with heterosexual orientation numerically constituted a minority, which illustrates the general change of HIV infection channels observed not only in Europe but also worldwide^3,8^. Therefore, it can be assumed that, compared with the general population, it is not the *general* status of being in a sexual minority but being in a *relative* minority that may matter most to personal experience of HIV/AIDS stigma.

This finding adds a new perspective to the minority stress theory, which postulates that stigmatised groups (e.g. LGBT individuals) suffer from greater social stressors because of their minority social status in relation to the distribution of a given characteristic in the general population^17,23,39^. Thus, the question arises of whether our intriguing result can be attributed to the uniqueness of PLWH as a clinical sample^6,7^, the cultural context^40^ or an interaction of both^39^. That is, it has been shown in many reports that LGBT people often suffer from various forms of discrimination and harassment; in particular, they experience stigmatisation for being people outside ‘normal’ society, defined in terms of the majority norm^41–43^, which was even strengthened during the COVID-19 pandemic due to misinformation surrounding the pandemic linking COVID-19 to HIV infection^30^. They are also unfairly perceived by society as a group among which sexual transmitted diseases (e.g. HIV infection) are much more common in comparison with the mainly heterosexual general population^44^. Nevertheless, LGBT people constitute more than half of all PLWH in both the USA^45^ and Europe^46^, including the country in which the study was conducted^47^. Thus, although people with heterosexual orientation constitute the majority in the general population, they may be a minority within the PLWH population; therefore, when they become infected with HIV, they may experience a double source of stress. Firstly, they have *illness outside from a normal community*, which was how HIV/AIDS was known when it emerged^48^, and this is still how it is perceived by society^7^. Secondly, they present a sexual orientation that is different to what has historically been related to the transmission of HIV infection^25^.

Nevertheless, the above-mentioned picture of our results becomes even more interesting when we look at the results of Hypothesis 2, evaluating co-occurrence of PLWH with heterosexual orientation versus other sexual orientations and their gender. Specifically, the highest level of total HIV/AIDS stigma is among heterosexual women, while for men there is no relationship between sexual orientation and stigma. This result is even further modified when being diagnosed with AIDS was added when testing Hypothesis 3; namely, this effect is present only for women being diagnosed with AIDS.

On the one hand, this result is consistent with the general trend in the literature indicating a much lower level of health-related quality of life among females living with HIV compared to males^49–51^, and more intense HIV/AIDS stigma, intimate partner violence, higher rate of associated trauma and mental problems in HIV-positive women^24^. It also corresponds with the so-called gender paradox, which demonstrates that in disorders with an unequal gender ratio, members of the gender with the lower prevalence rate tend to be more seriously affected in terms of comorbidity and poor health outcomes^52^. According to epidemiological data, in Europe, the prevalence rate for HIV-infected women is much lower compared with that of HIV-infected men (the overall male-to-female ratio was 3.2 in 2020), but their HIV-related burden is higher^3^; thus, being female represents a relative minority status among PLWH. Yet there is an accumulation of all three minority statuses (i.e. being female, heterosexual and diagnosed with AIDS) that differentiates this group in terms of total HIV/AIDS stigma from other combinations of these statuses. Although participants with co-occurrence of all these statuses reported the highest total stigma, pairwise comparisons were only significant for women with non-heterosexual orientations diagnosed with AIDS, who at the same time reported the *lowest* total stigma. In this light, the effect of a relative minority can be seen even more strongly; being a minority within a minority may be especially stigmatising, as someone may suffer from this stigmatisation from both representatives of the healthy general population and representatives of a group to which, according to the general population, someone belongs because of their health status. Still, being in a minority, but within this minority rooted in another community, may even have a protective effect, observed here only for women diagnosed with AIDS reporting other than heterosexual orientation.

Analysis of all the stigma subscales together shows significant effects only for disclosure stigma and only with regard to Hypotheses 2 and 3. Interestingly, either alone or in combination, the studied minority statuses have no effect on personalised stigma, negative self-image and concerns about public attitudes. Thus, only concern about revealing one’s HIV-infected status to someone else is prone to a combination of minority statuses. Several studies have shown that disclosure concern still constitutes the most sensitive and difficult issue for PLWH^6^. Moreover, for this particular area of stigma, the results differ slightly from the picture described for total stigma. Although the effect of sexual orientation and gender on disclosure is the same as on total stigma, the effect of the three-way interaction additionally reveals a significant effect also for men. This is opposite to what can be seen for women.

Specifically, as we have already discussed, women with heterosexual orientation diagnosed with AIDS experienced *higher* disclosure stigma than women with other sexual orientations diagnosed with AIDS, whereas for men those with heterosexual orientation diagnosed with AIDS experienced *lower* disclosure stigma than those with other sexual orientations diagnosed with AIDS. The contrast, however, was more pronounced for women than for men. Thus, the effects of the overlapping of the three minority statuses in HIV infection are different for women and men, not only in terms of direction but also strength. For women, AIDS status further magnifies the patterns regarding their disclosure concerns already observed in the context of their sexual orientation, while for men, AIDS status makes a substantial difference. It seems that for men, when it comes to disclosure concerns, being other than heterosexual and diagnosed with AIDS leads to an accumulation of distress. This finding may add to the literature on minority stress among male PLWH^19,22^ by pointing out that the combination of these two characteristics (i.e. sexual orientation and AIDS status), rather than each of them alone, may be strong enough for elevating HIV-related distress and taking precautions to prevent accidental disclosure^22^.

To sum up, when suffering from AIDS, women’s concerns regarding revealing HIV status are the highest among those who are heterosexually orientated, whereas for men, this is the case among those who are other than heterosexually oriented. The effect of accumulation of minority statuses on self-reporting stigma may thus depend on what group is taken for comparisons (inner or outer) and what areas of stigma are evaluated. Consequently, in terms of HIV stigma among PLWH, *relative* minority status effects were obtained for women, while *general* minority status effects were obtained for men.

### Strengths and limitations

This study has several strengths, including the large clinical sample observed during a critical time of the COVID-19 pandemic. Nevertheless, a few limitations should be underlined. First, the cross-sectional design precludes causal interpretations of the findings. Secondly, the study sample was homogenous with respect to HIV infection control (ART) and comprised mostly of highly functioning PLWH. Third, we did not control other than the studied minority statuses and being infected with HIV as sources of possible stigmatisation (for instance, populations at special risk include migrants and sex workers). Also, our sample was not large enough to analyse the whole spectrum of sexual orientations, and men having sex with men were not a separate category; thus, they may have identified themselves with any of them. Still, adding such a category might not bring more accurate classification due to either social desirability or denial. Lastly, due to ethical and legal issues regarding data protection (i.e. third party access to medical records) we based our analysis on self-reported clinical variables, with the exception of medical confirmation of HIV infection.

## Conclusion

The results showed that the among the studied sources of stigma, there is the effect of an accumulation of the minority statuses of PLWH, rather than the main effects of each status separately. Also, each minority status should be analysed from at least two perspectives, namely *general* (i.e. in comparison with the general population) and *relative* (i.e. in comparison with a given population). Both of these should be taken into an account in further studies on the HIV/AIDS stigma. In this light, from a theoretical perspective, understanding objective and perceived minority status is likely to be key in comprehensively elucidating the mechanisms through which stigma affects the well-being of PLWH and hampers efforts towards effective prevention^21,53^. On the other hand, from a clinical perspective, the results of our study have the potential to facilitate the development of a relatively simple screening tool to identify individuals who may be at risk of experiencing elevated levels of stigma due to HIV, particularly in relation to disclosure.

## Data Availability

All the data are available upon the request from the corresponding author.
